# Use of out-of-hospital cardiac arrest registries to assess COVID-19 home mortality

**DOI:** 10.1186/s12874-020-01189-3

**Published:** 2020-12-14

**Authors:** Hervé Hubert, Valentine Baert, Jean-Baptiste Beuscart, Emmanuel Chazard

**Affiliations:** 1 University of Lille, CHU Lille, ULR 2694 - METRICS: Évaluation des Technologies de Santé et des Pratiques Médicales, University of Lille, F-59000 Lille, France; 2 French National Out-of-Hospital Cardiac Arrest Registry, Lille, France; 3 Institute of Health Engineering of Lille, ULR 2694 – METRICS, University of Lille, 42 Rue Ambroise Paré, 59120 Loos, France

**Keywords:** COVID-19, Out-of-hospital cardiac arrest, COVID-19 home mortality, Epidemiology

## Abstract

**Background:**

In most countries, the official statistics for the coronavirus disease 2019 (COVID-19) take account of in-hospital deaths but not those that occur at home. The study’s objective was to introduce a methodology to assess COVID-19 home deaths by analysing the French national out-of-hospital cardiac arrest (OHCA) registry (RéAC).

**Methods:**

We performed a retrospective multicentre cohort study based on data recorded in the RéAC by 20 mobile medical teams (MMTs) between March 1st and April 15th, 2020. The participating MMTs covered 10.1% of the French population. OHCA patients were classified as probable or confirmed COVID-19 cases or as non-COVID-19 cases. To achieve our primary objective, we computed the incidence and survival at hospital admission of cases of COVID-19 OHCA occurring at home. Cardiac arrests that occurred in retirement homes or public places were excluded. Hence, we estimated the number of at-home COVID-19-related deaths that were not accounted for in the French national statistics.

**Results:**

We included 670 patients with OHCA. The extrapolated annual incidence of OHCA per 100,000 inhabitants was 91.9 overall and 17.6 for COVID-19 OHCA occurring at home. In the latter group, the survival rate after being taken to the hospital after an OHCA was 10.9%. We estimated that 1322 deaths were not accounted in the French national statistics on April 15, 2020.

**Conclusions:**

The ratio of COVID-19 out-of-hospital deaths to in-hospital deaths was 12.4%, and so the national statistics underestimated the death rate.

## Background

The severity of the coronavirus disease 2019 (COVID-19) is directly related to resulting health complications like severe acute respiratory syndrome, septic shock, sepsis, and cardiac, thrombotic or thromboembolic disorders [1–7]. These complications can lead to out-of-hospital cardiac arrest (OHCA) when patients are not rapidly admitted to hospital [8, 9]. The World Health Organization (WHO) and most national governments provided daily death COVID-19 counts. The COVID-19 mortality rates are difficult to estimate because the total number of infected patients in a given country will depend on the national or regional testing strategy. Moreoadditional

ver, most COVID-19 death counts are limited to in-hospital deaths, which is unlikely to account for all COVID-19 mortalities. At the beginning of April, the French Ministry of Health started to include care home deaths in the official statistics, which led to a marked jump in the total COVID-19-related death count in France. Many deaths at home are still not accounted for or even assessed in official statistics [10]. In many countries, the strategy for hospital-based COVID-19 management involves detecting high-risk patients who require hospitalization and specific care. Patients with few or mild symptoms may not be hospitalized, and are instructed to go to the emergency department only if their condition deteriorates [11]. However, healthcare professionals have noticed that a patient’s condition sometimes deteriorates very quickly. It is not known how often COVID-19 patients die as a result of an acute clinical deterioration that starts at home, i.e. before they are admitted to the emergency room or even before a mobile medical team (MMT) arrives. The present study’s objective was to introduce a methodology to assess the number of COVID-19 patients who die at home. To this end, we analysed data from the French national out-of-hospital cardiac arrest (OHCA) registry (RéAC) [12].

## Methods

### Study setting

France has a two-tiered system for prehospital emergency medical assistance. The first tier corresponds to a fire department ambulance for rapid intervention and the provision of basic life support (BLS). The second tier corresponds to a mobile emergency and resuscitation service (MERS) comprising one or more MMTs, which can provide advanced life support (ALS) if required [13]. Medical dispatch centers are responsible for out-of-hospital emergency coordination. During OHCA interventions, all MMTs used a specific Utstein-style RéAC form to document patient data, times, care provision, and immediate survival status [12, 14]. Lastly, the data are stored in the RéAC’s secure database (www.registreac.org).

### Time period and population

**Fig. 1:**
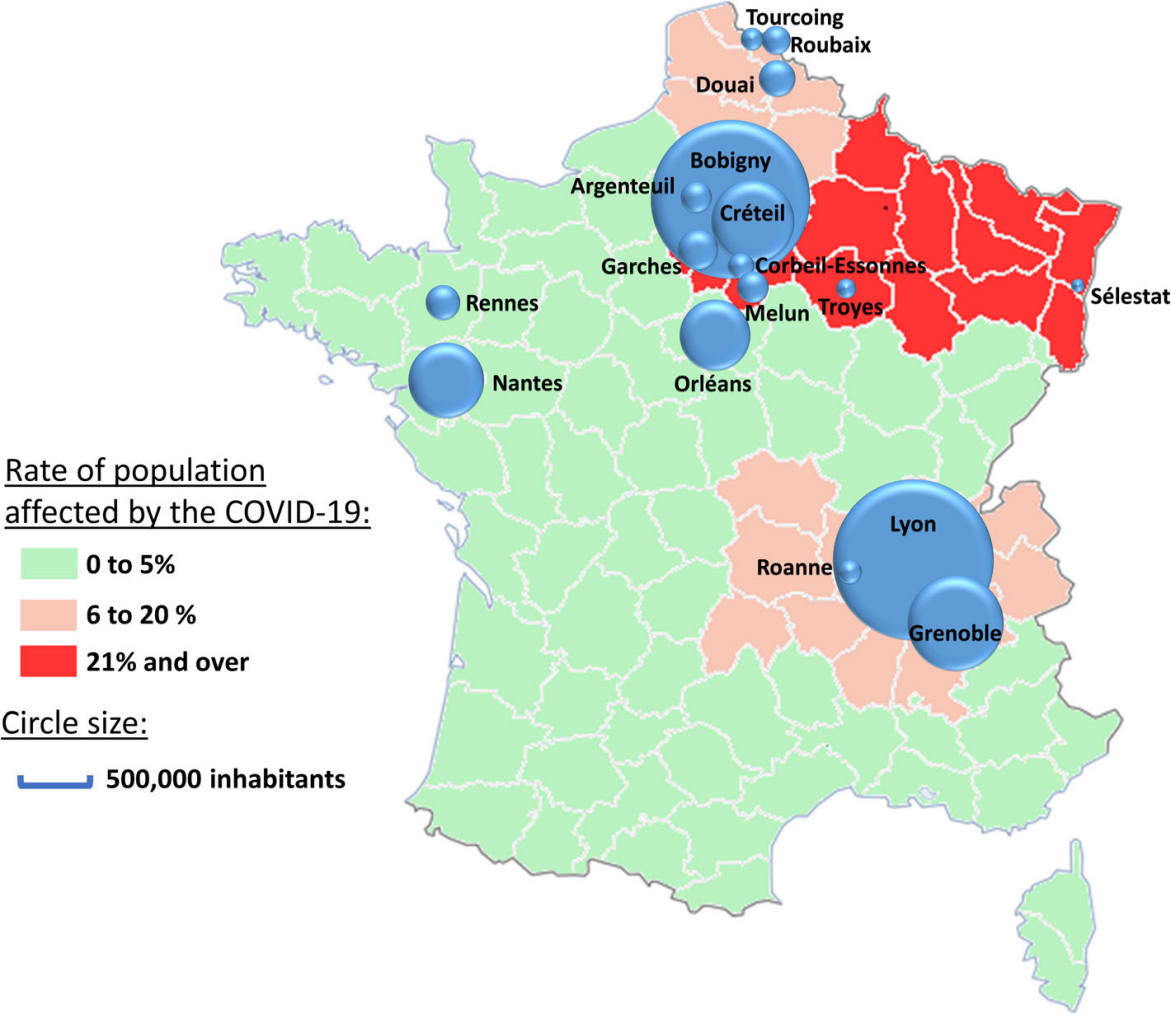
Participating centers, populations covered, and the rate of COVID-19 contamination on April 15th, 2020, by French administrative region. Bobigny comprises four MERSs (in Aulnay-sous-Bois, Bobigny, Montfermeil, and Saint-Denis). Original figures created by the authors

In this retrospective multicentre cohort study, we collected OHCA data, from 20 MERSs (Argenteuil, Aulnay-sous-Bois, Bobigny, Corbeil-Essonnes, Creteil, Douai, Garches, Grenoble, Lyon, Melun, Montfermeil, Nantes, Orléans, Rennes, Roanne, Roubaix, Selestat, Saint-Denis, Tourcoing, and Troyes) in towns and cities throughout France (Fig. 1) during a 46-day study period (from March 1st to April 15th, 2020). Hence, these centres covered an at-risk population of 6,793,921 inhabitants in Paris, its suburbs and in rural and urban towns of all sizes elsewhere in France. The study population was representative of the French population as a whole. We selected these 20 MERSs because we had checked that they included all their OHCAs in the RéAC; the data were always quality-controlled and the patient’s COVID-19 stats was always recorded. Trauma-related OHCAs were excluded. All the data (including the patient’s COVID status) were collected prospectively. Individuals with OHCA were classified as probable or confirmed COVID-19 cases or as non-COVID-19 cases as defined by the World Health Organization [15].

A probable case was defined as “a suspect case for whom testing could not be performed for any reason or for whom testing for the COVID-19 virus is inconclusive”. In this context, patients had:

- consulted a physician because of COVID-19 symptoms.
- met WHO criteria A, B or C (acute respiratory illness AND (A) a history of travel to or residence in a location reporting community transmission of COVID-19 disease during the 14 days prior to symptom onset or (B) having been in contact with a confirmed or probable COVID-19 case in the last 14 days prior to symptom onset or (C) in the absence of an alternative diagnosis that fully explains the clinical presentation) [15].
- not received a laboratory confirmation of COVID-19 but were considered as probable cases. Indeed, little testing was performed in France during the study period, and the tests that were performed were mainly limited to in-hospital and severe cases of COVID-19.

A confirmed case is defined by the WHO as “a person with laboratory confirmation of COVID-19 infection, irrespective of clinical signs and symptoms”. Hence, patients with laboratory-confirmed COVID-19 (after in-hospital or outpatient screening) and allowed to return home or remain at home (because of low severity) were considered as confirmed cases.

All other patients were included in the non-COVID-19 group.

This information was collected prospectively or gathered (after an auscultation) by the MMT’s emergency physician. We pooled probable and confirmed COVID-19 cases into a COVID-19 OHCA group.

### Statistical analysis and incidence assessment

Quantitative variables were expressed as mean ± standard deviation (SD). Categorical variables were expressed as number of cases and percentage. COVID-19 group and Non COVID-19 groups were compared. For quantitative variables, we used a Student t test and the result was reported as the mean difference, and its 95% confidence interval. For qualitative variable, we used a Fisher’s exact test, and its result was reported as the odds ratio (computed using the conditional maximum likelihood estimate) and its 95% confidence interval (CI).

To calculate incidence, we used data published in 2020 by the French National Institute of Statistics and Economic Studies (INSEE). These incidences were adjusted for age (in age classes: 0–29; 30–49; 50–65; 65–75; > 75 years) and sex. We computed the overall annual age- and sex-adjusted incidence of OHCA, the incidence in the non-COVID-19 OHCA group, and the incidence in the COVID-19 OHCA group. Lastly, to achieve our primary objective, we computed the incidence and survival at hospital admission in the “COVID-19 OHCA at home” group. The latter group did not include cases of CA that occurred in retirement homes or in public places, which were already accounted for in the French national statistics.

All statistical analyses were performed with SPSS software (version 25.0; IBM, Armonk, NY, USA). The threshold for statistical significance was set to *p* <  0.05.

## Results

We analysed 670 OHCAs. Of these, 146 were categorized into the COVID-19 group (127 probable cases and 19 confirmed cases). The mean age of the overall study population was 68.2 ± 17.2, with no difference between the COVID-19 and non-COVID-19 groups (difference and 95% CI: − 0.90 [− 4.09;2.23], *p* = 0.565). The population was predominantly male (68.8%), and there was a significant difference between the COVID-19 and non-COVID-19 groups (60.3% vs. 71.2% males, respectively; odds ratio and 95% confidence intervals (OR [95%CI]) 0.61 [0.41;0.92], *p* = 0.015). Most OHCAs occurred at home (85.2%), with no significant difference between the groups in this respect (OR [95%CI] 1.61 [0.92;3.03], *p* = 0.099). A history of diabetes was more frequent in the COVID-19 group than in the non-COVID-19 group (21.2% vs. 13.0%, respectively; OR [95%CI] 1.82 [1.09;2.94], *p* = 0.017). The two groups differed with regard to the causes of the OHCAs; relative to the COVID-19 group, the frequency of cardiac causes was higher in the non-COVID-19 group (34.9% vs. 72.9%, respectively; OR [95%CI] 0.20 [0.13;0.30], *p* <  0.001) and the frequency of respiratory causes was lower (54.1% vs. 9.9%, respectively; OR [95%CI] 11.11 [6.67;16.67], *p* <  0.001). There were no differences between the COVID-19 and non-COVID-19 groups with regard to the frequency of survival at hospital admission (10.3% vs. 13.9%, respectively; OR [95%CI] 0.71 [0.36;1.30], *p* = 0.271). These results correspond to 131 out the 146 deaths in the COVID-19 group and 451 of the 524 deaths in the non-COVID-19 group. The characteristics are detailed in Table 1.

**Table 1:** Description and comparison of the COVID-19 OHCA and non-COVID-19 OHCA in France from March 1st to April 15th, 2020

	COVID-19 OHCA (***N*** = 146)	Non-COVID-19 OHCA (***N*** = 524)	Estimated effect size^a^	***p***
**Context of the OHCA**				
Age - yr	67.5 ± 17.5	68.4 ± 17.1	−0.90 [−4.09;2.23]	0.565
Sex (male) - no. (%)	88 (60.3%)	373 (71.2%)	0.61 [0.41;0.92]	0.015
OHCA at home - no. (%)	129 (88.4%)	432 (82.4%)	1.61 [0.92;3.03]	0.099
Medical history - no. (%)				
- diabetes	31 (21.2%)	68 (13.0%)	1.82 [1.09;2.94]	0.017
- cardiovascular	60 (41.1%)	228 (43.5%)	0.91 [0.61;1.33]	0.637
- respiratory	23 (15.8%)	69 (13.2%)	1.23 [0.70;2.08]	0.417
- other	45 (30.8%)	151 (28.8%)	1.10 [0.72;1.67]	0.681
- unremarkable	9 (6.2%)	29 (5.5%)	1.12 [0.46;2.50]	0.840
Cause of the OHCA - no. (%)				
- cardiac cause	51 (34.9%)	382 (72.9%)	0.20 [0.13;0.30]	< 0.001
- respiratory cause	79 (54.1%)	52 (9.9%)	11.11 [6.67;16.67]	< 0.001
- other cause	16 (11.0%)	90 (17.2%)	0.60 [0.31;1.06]	0.073
Bystander presence (at collapse) - no. (%)	94 (64.8%)	279 (53.6%)	1.59 [1.06;2.38]	0.018
- Immediate bystander BLS^b^ - no. (%)	48 (52.7%)	139 (49.6%)	1.05 [0.64;1.72]	0.631
**Resuscitation**				
Bystander BLS - no. (%)	67 (45.9%)	237 (45.2%)	1.03 [0.70;1.51]	0.925
Bystander AED use - no. (%)	5 (6.3%)	32 (11.3%)	0.55 [0.16;1.45]	0.215
- if AED used, shock - no. (%)	1 (20.0%)	7 (24.1%)	0.89 [0.02;11.11]	0.999
BLS by first aid providers - no. (%)	119 (81.5%)	405 (77.4%)	1.30 [0.80;2.13]	0.309
ALS by the MMT - no. (%)	83 (56.8%)	312 (59.5%)	0.89 [0.61;1.32]	0.569
First recorded cardiac rhythm^c^ - no. (%)				
- asystole	122 (83.6%)	436 (83.4%)	1.03 [0.62;1.75]	0.999
- VF/pulseless VT	3 (2.0%)	35 (6.7%)	0.29 [0.06;0.95]	0.041
- PEA	15 (10.3%)	36 (6.9%)	1.56 [0.76;3.03]	0.215
- ROSC during BLS	6 (4.1%)	16 (3.1%)	0.94 [0.54;1.58]	0.599
**Times**				
Time between T_0_ and MMT arrival - min	30.4 ± 53.5	23.8 ± 24.6	6.60 [−2.56;15.60]	0.158
Time between T_0_ and ROSC or death - min	50.8 ± 52.4	42.0 ± 27.7	8.80 [−0.27;17.88]	0.057
**Survival**				
ROSC - no. (%)	23 (15.8%)	87 (16.7%)	0.94 [0.54;1.59]	0.900
Survival at hospital admission - no. (%)	15 (10.3%)	73 (13.9%)	0.71 [0.36;1.30]	0.271

*COVID-19* SARS-CoV-2 coronavirus; *OHCA* Out-of-hospital cardiac arrest; *BLS* Basic life support; *AED* Automated external defibrillator; *MMT* Mobile medical team; *ALS* Advanced life support; *VF* Ventricular fibrillation; *VT* Ventricular tachycardia; *PEA* Pulseless electrical activity; *ROSC* Return of spontaneous circulation

^a^ Odds ratio and 95% CI (Confidence Interval) for categorical variables, mean difference and 95% CI for quantitative variable

^b^if the OHCA was witnessed by a bystander

^c^on arrival of the MMT

The equivalent overall annual age- and sex-adjusted incidence of OHCA was 91.9 per 100,000 inhabitants. The equivalent annual age- and sex-adjusted incidences in the non-COVID-19 OHCA group and the COVID-19 OHCA were respectively 72.0 and 19.9 per 100,000 inhabitants. In the group of COVID-19 OHCAs occurring at home (*n* = 129), the equivalent annual incidence was 17.6 per 100,000 inhabitants; for France as a whole, this would correspond to 1483 OHCAs during the 46-day study period. The survival rate at hospital admission in the COVID-19 OHCA group was 10.9%.

## Discussion

During the first 6 weeks of the COVID-19 epidemic in France, we detected an increase in the overall incidence of OHCA of 30.4/100,000 inhabitants. We observed about 50% more OHCAs than had previously reported for France by Luc et al. [16] (61.5 per 100,000 inhabitants per year). This increase is in line with Marijon et al.’s results [17] in their regional study (Paris and its suburbs).

Of these 30.4 cases of OHCA per 100,000 inhabitants, 17.6 could be attributed to COVID-19 - corresponding to two thirds of the increase in incidence. When OHCA was related to COVID-19, the most frequently cause was a respiratory syndrome (in 50% of cases), followed by a cardiac cause. This increase in respiratory aetiologies might be directly linked to the rapid development of complications such as severe acute respiratory syndrome or cardiac and thromboembolic distress [2, 18]. Using the survival rate (10.9%) and the calculated number of cases of COVID-19 OHCA occurring at home (1483), we estimated that 1322 people in France died at home from COVID-19 over the study period. This number corresponds to one eighth of France’s official COVID-19 death count (which covered only in hospital deaths) for the same period (*n* = 10,643).

The increase in OHCA was also related to non-COVID-19 patients. The RéAC data do not indicate the reason for this increase. Nevertheless, an assumption could be that the prevention of healthcare and/or the chronic disease patients follow up might have been delayed during this pandemic. Indeed, healthcare resources was in majority dedicated to the COVID-19 management [19] and patients with chronic diseases might have dropped out or postponed their follow up [20]. We also hypothesize that some of the increase in the incidence of OHCA is also related to the pandemic situation (novel infections, low levels of testing, lockdown, no changes in the care procedure, more people waiting at home until a fatal deterioration occurred, etc.). These results are consistent with the INSEE study showing an increase 26% of deaths in France between early March and mid-April 2020 [21].

In the overall population (i.e. COVID-19 and non-COVID-19 OHCA patients), the survival at hospital admission rate in our study was very low (13.1%), relative to the value of 21% observed outside a pandemic period [22]. The same trend was observed for the ROSC rate: 16.5% in our study vs. 24.0% in the literature [22]. These lower survival rates might not have been related to COVID-19 because there were no significant differences between the COVID-19 and non-COVID-19 groups. The main factors associated with survival in OHCA (such as patient age or intervention time) were much the same as those previously reported before the pandemic [12, 22, 23]. Only a history of diabetes (a poor prognostic factor for COVID-19) was slightly more prevalent [24, 25]. When we looked for significant differences between COVID-19 and non-COVID-19 populations, the results highlighted some of the COVID-19 patients’ characteristics. Indeed, outside of a pandemic period, one usually observes more men, less bystander presence at the time of collapse, more cardiac causes, less respiratory causes, and a greater frequency of shockable rhythms. With regard to these variables, OHCA victims look more like COVID-19 patients than “usual” OHCA victims. The lower survival rates in the COVID-19 group might also be related (at least in part) to how the OHCAs were managed. Our results suggest that the proportion of OHCA patient receiving BLS or ALS from the professional first responders (the fire department or the MMT) was lower than usual. On the whole population, the fire brigade provided BLS in 78.3% of OHCAs, and the MMT provided ALS in 59.0% of OHCA. These rates are usually around 85 and 70%, respectively [22, 23]. At the start of the COVID-19 pandemic, there was a lack of clear guidelines on resuscitation. The WHO lists CPR as an aerosol-generating technique [26], whereas Couper et al.’s [27] recent systematic review could not establish whether or not chest compressions or defibrillation were associated with aerosol generation. In the West Midlands region of the UK, health care teams have been given instructions for the non-resuscitation of patients with suspected or confirmed COVID-19 [28]. This type of guideline was not in force in France. However, French professional first responders (i.e. fire department teams and MMTs) may have been more selective about deciding to resuscitate in this context.

The present study also had some limitations. We did not included data from all French regions. However, included centres came from several different areas (rural or urban) and there was a well balanced between strongly and less impacted by the COVID-19 regions. We classified the aetiologies of OHCA according to Ustein style. However, it is known that differentiating between cardiac and non-cardiac causes is difficult in a pre-hospital setting (as highlighted by Carter and Cone [29]). The very limited access to COVID-19 tests in France during the study period might have prompted us to under- or over-diagnose cases of COVID-19. During this period, auscultation was the most reliable way to determine the patient’ COVID-19 status in a prehospital setting. However, in the event of a second epidemic wave, with the current greatest availability of COVID-19 tests, we can now consider the generalization of prehospital RT-PCR tests in order to better identify COVID-19 cases.

## Conclusions

In summary, at-home COVID-19-related deaths corresponded to one eighth of in-hospital deaths, which means that 1322 deaths were not accounted for by the French national statistics as of April 15th, 2020. This finding should prompt us to consider improving the triage of COVID-19 patients, in order to improve care strategies, monitor the disease more accurately, and limit home mortality due to the virus. Many countries (e.g. Germany, England, Italia, USA, Canada, Spain, Japan, Australia, Singapore, etc.) have developed their own national OHCA registry. These countries could therefore replicate our methodology and assess their at-home COVID-19 mortality rate.

## Data Availability

Anonymized cardiac arrest data can be requested from scientific committee of the French Cardiac Arrest Registry (RéAC – www.registreac.org). Demographic data are available online in the public domain from the French National Institute of Statistics and Economic Studies (INSEE – https://www.insee.fr/fr/statistiques?theme=0).

## Abbreviations

OHCA: Out-of-hospital cardiac arrest
WHO: World health Organization
MERS: Mobile emergency and resuscitation service
MMT: Mobile medical team
RéAC: French national out-of-hospital cardiac arrest registry
INSEE: French national institute of statistics and economic studies
BLS: Basic life support
ALS: Advanced life support
